# Optimized Method for Untargeted Metabolomics Analysis of MDA-MB-231 Breast Cancer Cells

**DOI:** 10.3390/metabo6040030

**Published:** 2016-09-22

**Authors:** Amanda L. Peterson, Adam K. Walker, Erica K. Sloan, Darren J. Creek

**Affiliations:** 1 Drug Delivery, Disposition and Dynamics, Monash Institute of Pharmaceutical Sciences, Monash University, Parkville, Victoria 3052, Australia; Amanda.peterson@monash.edu; 2 Drug Discovery Biology, Monash Institute of Pharmaceutical Sciences, Monash University, Parkville, Victoria 3052, Australia; Adam.walker@monash.edu (A.K.W.); erica.sloan@monash.edu (E.K.S.); 3 Division of Cancer Surgery, Peter MacCallum Cancer Centre, Melbourne, Victoria 3000, Australia; 4 Cousins Center for PNI, University of California Los Angeles, Los Angeles, CA 90095, USA; 5 UCLA Semel Institute, University of California Los Angeles, Los Angeles, CA 90095, USA; 6 Jonsson Comprehensive Cancer Center, University of California Los Angeles, Los Angeles, CA 90095, USA; 7 UCLA AIDS Institute, University of California Los Angeles, Los Angeles, CA 90095, USA

**Keywords:** cell metabolomics, breast cancer, glucose, glutamine, isotope labelling

## Abstract

Cancer cells often have dysregulated metabolism, which is largely characterized by the Warburg effect—an increase in glycolytic activity at the expense of oxidative phosphorylation—and increased glutamine utilization. Modern metabolomics tools offer an efficient means to investigate metabolism in cancer cells. Currently, a number of protocols have been described for harvesting adherent cells for metabolomics analysis, but the techniques vary greatly and they lack specificity to particular cancer cell lines with diverse metabolic and structural features. Here we present an optimized method for untargeted metabolomics characterization of MDA-MB-231 triple negative breast cancer cells, which are commonly used to study metastatic breast cancer. We found that an approach that extracted all metabolites in a single step within the culture dish optimally detected both polar and non-polar metabolite classes with higher relative abundance than methods that involved removal of cells from the dish. We show that this method is highly suited to diverse applications, including the characterization of central metabolic flux by stable isotope labelling and differential analysis of cells subjected to specific pharmacological interventions.

## 1. Introduction

Cancer is a disease state in which cellular processes—including metabolism—are altered, contributing to uncontrolled cell proliferation. The dysregulation of cellular respiration, which is common in cancer cells, is better known as the Warburg effect or aerobic glycolysis, whereby cells metabolize glucose via glycolysis in preference to oxidative phosphorylation as the primary means of energy generation [1,2]. Glutamine is another key nutrient that plays a pivotal role in metabolic processes within proliferating tumour cells, including production of energy for biological processes, protection against oxidative stress and providing both carbon and nitrogen sources for macromolecule synthesis [3,4,5]. In addition to these central metabolic pathways, altered amino acid, lipid and nucleotide metabolism has been associated with cancer cells, and cellular metabolism is seen as a key target for the diagnosis and treatment of specific cancers [6,7,8,9,10].

Metabolomics is a useful tool to probe cancer cell metabolism, and offers insight into how metabolic processes may influence cancer progression. Metabolomics strategies typically employ either a hypothesis-driven approach or a hypothesis-forming approach. The hypothesis-driven method involves assessing either a specific pathway or metabolites of interest, based on previous knowledge of the involvement of that pathway or metabolite in the phenotype or intervention under investigation. In contrast, an untargeted or hypothesis-forming approach allows for an unbiased investigation of all metabolic pathways under defined experimental conditions. To facilitate these approaches, a number of analytical techniques may be used to detect changes in metabolite levels, including nuclear magnetic resonance (NMR), gas chromatography mass spectroscopy (GC-MS) and liquid chromatography mass spectroscopy (LC-MS). There are advantages and limitations to each analytical method. The key advantages of NMR are that it is inherently quantitative, highly reproducible, and can provide structural information which enables accurate identification of metabolites. However, when comparing this technique to other analytical platforms, it is less sensitive than MS and is capable of detecting only a limited number of metabolites. Comparatively, GC-MS is useful for the detection and identification of a range of metabolites, and comprehensive spectral databases are available for metabolite identification. However, GC-MS requires derivatization of most metabolites to make them volatile, and if compounds are not volatile and unable to be derivatized then they will not be detected. Lastly, LC-MS combines the separation capabilities of chromatography with the sensitivity of MS, without the need to derivatize samples, allowing detection and identification of a wide range of metabolites with high sensitivity from a complex sample. These characteristics have led to the widespread application of LC-MS for metabolomics studies, and make it particularly suitable for untargeted analyses [11,12].

The main aim of this study was to develop a method for the optimal detection of metabolites from MDA-MB-231 cells using LC-MS. This methodology is suitable for untargeted metabolomics analysis of breast cancer cells and is capable of providing a better understanding of the relationships between cancer cell metabolism and proliferation, metastasis, or the impact of various exogenous and endogenous stimuli (e.g., drugs, stress, nutrients). MDA-MB-231 cells are triple negative breast cancer cells which lack oestrogen, progesterone and human epidermal growth factor receptor 2 (HER2) receptors, thus providing significant challenges for therapy [13]. As such, triple negative breast cancer is associated with poor prognosis [14,15]. It is known that breast cancer cells have altered central carbon metabolism and lipid metabolism, therefore developing a method that extracts both polar and non-polar metabolites will allow a global analysis of metabolism [9]. The MDA-MB-231 cell line is routinely used in both in vivo and in vitro studies to investigate cancer progression [16]. Despite the widespread use of this cell line, a reliable and sensitive protocol for an untargeted extraction of metabolites has not been reported for this cell line. Current adherent cell extraction methods may not be ideally suited to our aims for several reasons: Methods that had been optimized for alternative analytical platforms were not suitable for untargeted LC-MS analysis [17,18,19,20,21,22,23]. Additionally, extraction methods for different mammalian cell lines may not be ideal for MDA-MB-231 cells due to the different metabolic profile and physical properties of each cell type [24]. Methods for detaching adherent cells from culture dishes using trypsin have been shown to cause significant metabolite leakage [23,24,25]. Lastly, methods developed for targeted analyses are not suitable for untargeted analyses as they are optimized to extract metabolites from a specific metabolite class (e.g., only polar metabolites) [26,27,28].

In this study, we have developed and validated an untargeted metabolomics method that was able to extract, detect and provide relative quantification for hundreds of lipid and polar metabolites in a monophasic extract from MDA-MB-231 cells using LC-MS analysis. The application of this methodology was demonstrated by addition of stable isotope labelled glucose and glutamine to reveal flux in central metabolic pathways, and by pharmacological intervention studies.

## 2. Materials and Methods

### 2.1. Materials

High glucose Dulbecco’s Modified Eagle Medium (DMEM), with Glutamax supplement and pyruvate was purchased from Invitrogen (Thermo Fisher Scientific, Waltham, MA, USA). Foetal bovine serum (FBS) was purchased from Invitrogen. (−)-isoproterenol hydrochloride (isoproterenol) and (*S*)-(−)-propranolol hydrochloride (propranolol) were purchased from Sigma Aldrich (St. Louis, MO, USA). For the isotope labelling studies, U-^13^C_6_-d-Glucose was purchased from Sigma Aldrich and U-^13^C_5_-l-Glutamine was purchased from NovaChem. All other solvents and reagents were of LC-MS analytical grade (Merck, Kenilworth, NJ, USA).

### 2.2. Cell Culture

MDA-MB-231^HM^, a highly metastatic variant of the MDA-MB-231 triple negative breast adenocarcinoma cell line, were a kind gift from Dr Zhou Ou (Fudan University, Shanghai Cancer Center, Shanghai, China) and were karyotyped for confirmation (Cellbank, Westmead, Australia) [29,30,31]. Cells were grown in DMEM, supplemented with 10% FBS, at 37 °C and 5% CO_2_.

### 2.3. Sample Preparation for LC-MS Analysis

**Figure 1:**
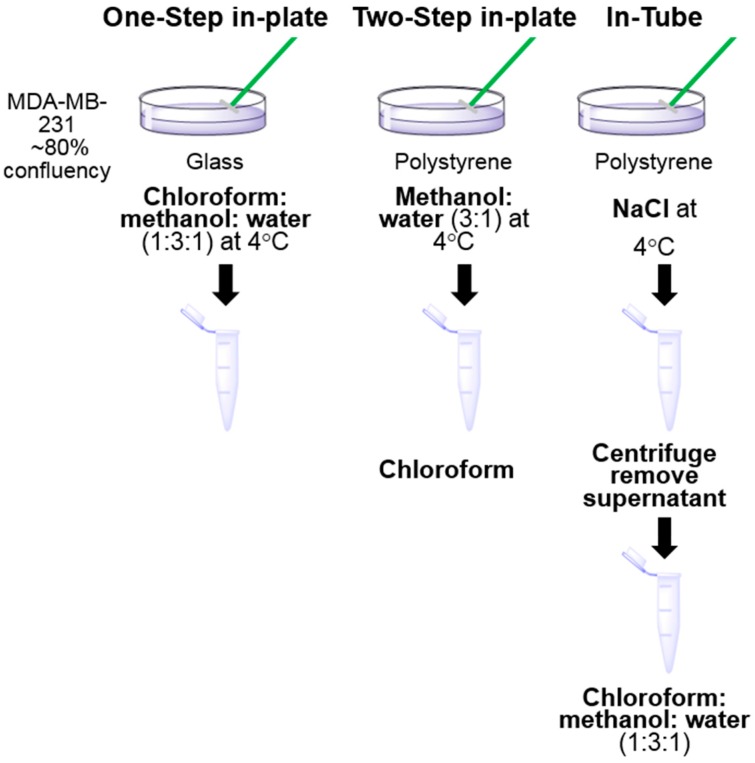
Workflow of metabolite extraction methods. For the one-step in-plate method, MDA-MB-231 cells grown in glass dishes were scraped in chloroform:methanol:water (1:3:1) for simultaneous extraction of polar and lipid metabolites. For the two-step in-plate method, cells were scraped in methanol:water (3:1) and chloroform was added after transfer of the mixture to a tube. For the in-tube method, cells were scraped in saline and transferred to tubes, where they were extracted in chloroform:methanol:water (1:3:1).

Three monophasic extraction methods were compared for metabolomics analysis (Figure 1). For the “one-step in-plate” extraction method, 10^6^ cells were grown in 10 cm glass dishes (Corning) coated with 10 mM fibronectin. For all other methods, 10 cm polystyrene cell culture dishes (Falcon) were used. Cells were harvested at 70%–80% confluency and all experiments were performed in replicate with cultures prepared and extracted on separate days within a 2-week period (*n* = 3–4).

For all extractions, immediately prior to cell harvest, cells were quenched and washed three times with 4 °C 0.9% NaCl (Baxter, Sydney Australia), and all subsequent steps in the extraction process were performed at 4 °C in a cold room. For the “in-tube” extraction method, cells were scraped in 0.5 mL 0.9% NaCl in water (4 °C), centrifuged at 200 × *g* for 5 min at 4 °C and resuspended in 200 µL of extraction solvent (chloroform:methanol:water = 1:3:1) at 4 °C. For the “two-step in-plate” extraction method, cells were scraped in 600 µL ice cold extraction solvent (methanol:water = 3:1), transferred to a 1.5 mL Eppendorf tube and 150 µL chloroform was added. For the “one step in plate” extraction method, cells were scraped in 750 µL of ice cold extraction solvent (chloroform:methanol:water = 1:3:1). After scraping, all extraction samples were mixed thoroughly on a vortex mixer for 30 min at 1200 rpm at 4 °C, then centrifuged at 20,000 × *g* for 10 min at 4 °C. For the “in-tube” extraction method, the supernatant (160 µL) was transferred into a glass vial for LC-MS. All other samples were evaporated to dryness under a nitrogen stream. All samples were then frozen at −80 °C until LC-MS analysis.

### 2.4. Stable Isotope Labelling and Drug Treatment

10^6^ cells were seeded in fibronectin-coated glass dishes. After 44 h in culture, cells were treated with 1 µM isoproterenol (or vehicle) for 3 h and then the medium was replaced with medium containing 25 mM U-^13^C_6_-d-Glucose labelled medium, or 1 mM U-^13^C_5_-Glutamine labelled medium (or isoproterenol as appropriate). After 1 h, samples were processed for LC-MS as described above. To determine specificity, some samples were treated with 5 µM propranolol for 30 min before addition of isoproterenol. The glucose-labelled media was prepared by adding the stable-isotope labelled glucose into DMEM to give a 50:50 ratio of U-^13^C and U-^12^C d-glucose. The glutamine-labelled media was prepared by adding the stable-isotope labelled glutamine into DMEM without l-glutamine or Glutamax to give 100% U-^13^C l-glutamine.

### 2.5. LC-MS Analysis

Before analysis, samples were thawed and reconstituted in 160 µL (chloroform:methanol:water = 1:3:1) with vortex mixing, followed by centrifugation to remove any precipitate. One hundred fifty microlitres of supernatant was transferred into a LC-MS glass vial. A quality control (QC) sample was prepared by pooling 10 µL from each vial. Samples were analysed on a Q-Exactive Orbitrap MS (Thermo Fisher Scientific, Pleasanton, CA, USA) with the use of hydrophilic interaction chromatography in both positive and negative modes. This was done using a ZIC-pHILIC 150 mm × 4.6 mm, 5 µm column (Merck Sequant, Darmstadt, Germany), which was coupled to a U3000 RSLC HPLC (Dionex, Thermo Fisher Scientific, Germering, Germany) The mobile phase (A) consisted of 20 mM ammonium carbonate in Milli-Q water and mobile phase (B) was comprised of 100% acetonitrile. The method was run as a gradient: 0 min 80% B; 15 min 50% B; 18 min 5% B; 24 min 80% B at a flow rate of 0.3 mL/min. The total run time was 32 min per sample. The samples were placed into the autosampler where they were maintained at 4 °C and 10 µL sample was injected onto the column, which was kept at 25 °C. Mass spectrometry was performed using a Q-Exactive Orbitrap, which was operated in polarity switching mode, with the following settings: resolution 35,000, AGC 1 × 10^6^, *m*/*z* range 85–1275, sheath gas 50, auxiliary gas 20, sweep gas 2, probe temperature 150 °C, and capillary temperature 300 °C. For positive mode ionization: source voltage +4 kV, S-lens voltage +50 V. For negative mode ionization: source voltage −3.5 kV, S-lens voltage −50 V. A mass calibration was performed for each polarity before running each batch to ensure accurate masses. Over 200 authentic metabolite standards were analysed at the start of each batch to provide accurate retention times to facilitate metabolite identification. Samples were analysed in random order with periodic injections of the pooled QC sample, and blank samples, throughout the batch.

### 2.6. LC-MS Data Processing

The raw metabolite data were processed using XCMS (Centwave) software for peak picking and mzMatch.R software for alignment and annotation of related metabolite peaks. Metabolites were then identified in IDEOM software, version 19c by matching the mass of each peak and its retention time with a database, using a mass accuracy window of 2 ppm and a retention time window of 5% for metabolites matching authentic standards, and 35% for other putative metabolites based on a retention time prediction model [32]. Noise and mass spectrometry artefacts were filtered using previously described algorithms [32,33] to minimize false identifications. Detection of stable isotope labelled metabolite peaks was performed using mzMatch-ISO [34]. Initial statistical analysis of metabolomics data was performed with IDEOM (Excel) using peak intensities (height) for all detected putative metabolites. Further analysis was undertaken using Tracefinder, version 3.1 (Thermo) to obtain manually curated accurate peak areas for selected analytes to allow targeted univariate analyses for metabolites in key pathways.

### 2.7. Statistical Analysis

All data are presented as mean ± SE. Statistical differences were determined using one-way ANOVAs and Dunnett’s or Tukey’s multiple comparisons where significant interactions were observed. Significance was determined at *p* values <0.05. Univariate analyses used GraphPad Prism software, version 6.05 (GraphPad Software).

## 3. Results

### 3.1. One-Step In-Plate Extraction Method Is Optimal for MDA-MB-231 Untargeted Metabolomics

To develop a robust technique to prepare extracts of MDA-MB-231 cells for metabolomics analysis, three different extraction methods were tested; a “one-step in-plate” extraction, a “two-step in-plate” extraction method, and an “in-tube” extraction method (Figure 1). These methods vary in how metabolism is quenched and metabolites are extracted. The “in-tube” extraction method quenches metabolism first, allowing transfer of intact cells from the plate to a tube by physical scraping, followed by a solvent-based metabolite extraction. In the “one-step in-plate method” the extraction solvent is added directly to the plate, ensuring simultaneous quenching and extraction of all metabolites. As efficient extraction of lipid metabolites can be enhanced with chloroform [24,28], which is incompatible with polystyrene cell culture dishes, cells were grown in glass dishes for this method. However, as many labs routinely grow cells in polystyrene dishes, we also investigated a “two-step in-plate method”, in which metabolism was quenched and polar metabolites extracted with 75% methanol in water, followed by a second step involving transfer to a microfuge tube and addition of chloroform to enhance extraction of lipid metabolites.

**Figure 2:**
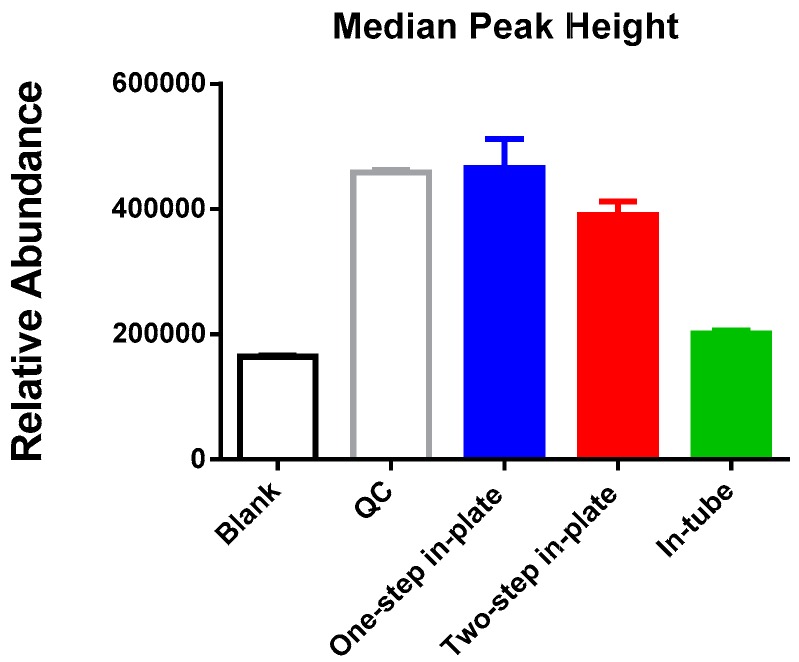
Comparison of global metabolite extraction efficiency: MDA-MB-231 cells were extracted using the “one-step in-plate”, ” two-step in-plate” or “in-tube” protocol (see Figure 1) and analysed on an LC-MS untargeted metabolomics platform. *y-*axis (relative abundance) represents the median peak height from all detected LC-MS peaks (mean ± SE; *n* = 3–4).

**Figure 3:**
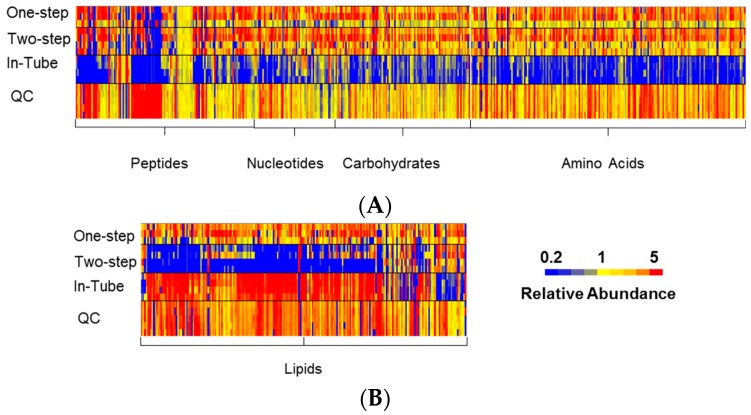
Heat map of relative abundance of metabolites detected for each sample from the three extraction methods: Putatively identified metabolites clustered according to pathway/class (from Ideom database) and relative abundance shown as LC-MS peak height normalized to the mean for each metabolite. Heat map shows low relative abundance (blue) of polar metabolites (**A**) and high relative abundance (red) of lipids (**B**) for the “in-tube” method. Low recovery (blue) of lipid metabolites (**B**) is evident for the “two-step in-plate” method. Minimal variance observed within the quality control (QC) samples (all five replicates shown) confirms the excellent technical precision of the LC-MS method.

The “in-tube” extraction method performed poorly, with few metabolites reliably detected, as indicated by the median LC-MS peak height from all detected features, which was only marginally greater than that seen in solvent blanks (Figure 2). The median LC-MS peak height was significantly higher in both of the two in-plate extraction methods, indicating significantly enhanced extraction efficiency (Figure 2). A qualitative analysis of the metabolite classes detected for each of the methods revealed no difference in the scope of polar metabolites detected for the two in-plate extraction methods (Figure 3A). However, the one step in-plate extraction method detected higher levels of lipid metabolites than the two-step in-plate extraction method (Figure 3B). Interestingly, the in-tube method performed very well for the detection of lipid species, despite being largely ineffective at extracting polar metabolites (Figure 3).

**Figure 4:**
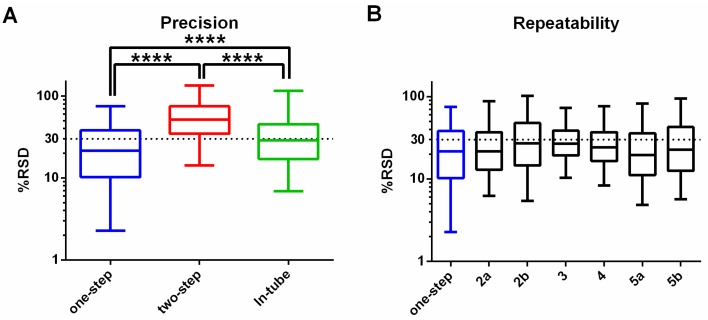
Precision and repeatability of the “One-step in-plate” method: The relative standard deviation (RSD) for each metabolite was determined for: (**A**) each of the extraction methods within a single experiment (*n* = 3–4); and (**B**) the “one-step in-plate” method performed on seven independent occasions (Note: experiments 2a and 2b were analysed in a single LC-MS batch, as were 5a and 5b). Box plots represent the median ± interquartile range, and whiskers show the 5th and 95th percentile (*n* = 3–4). **** *p* ≤ 0.0001, using one-way ANOVA with Tukey’s multiple comparison test.

In addition to metabolite coverage and sensitivity, quantitative reproducibility is a critical feature of any metabolomics workflow. The relative standard deviation (%RSD; %CV) was calculated for all detected putative metabolites in each of the three methods, revealing %RSD values less than 30% for 63% of putative metabolites detected in the “one-step in-plate” method. Precision was considerably worse for the “in-tube” and the “two-step in plate” methods (Figure 4A). The precision of the “one-step” method was further tested across six additional experiments (over four different occasions) to confirm reproducibility. The median %RSD was consistently below 30%, suggesting that this method is appropriate for semi-quantitative comparative studies (Figure 4B). These findings demonstrate that the “one-step” extraction method provides optimal detection of a broad range of metabolites, and has sensitivity and precision that is suitable for application in untargeted metabolomics studies.

### 3.2. Application 1: Determination of Metabolic Flux with Isotope Labelling

**Figure 5:**
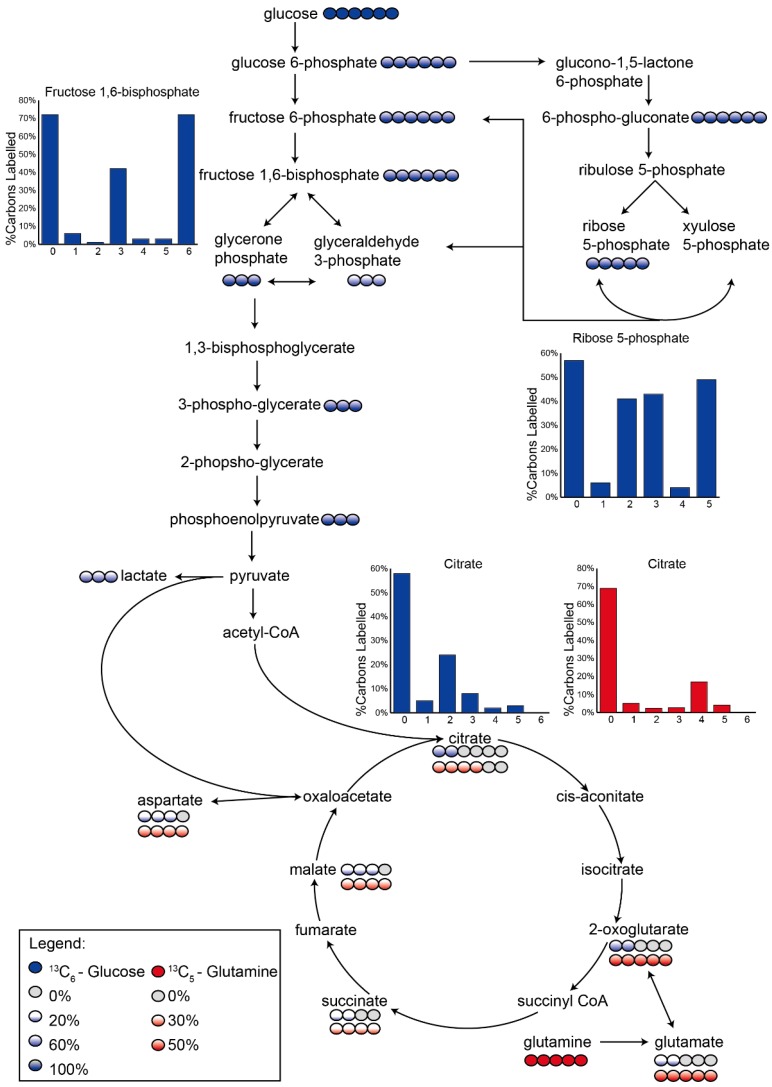
Schematic of central carbon pathways showing U-^13^C-glucose and U-^13^C-glutamine labelling. Incorporation of glucose (blue) or glutamine (red) into central carbon metabolism in MDA-MB-231 cells after 1 h. Circles represent the number of carbon atoms in each metabolite, with the shaded circles corresponding to the number of labelled carbons in the most abundant isotopologue. The shading intensity corresponds to the percentage of ^13^C-enrichment (darker = higher enrichment), with values from ^13^C-glucose labelling corrected (doubled) to allow for the 50% initial glucose enrichment. Column charts show relative isotopologue abundances for selected metabolites with complex labelling profiles (*x*-axis indicates the number of ^13^C-labelled carbons in each isotopologue).

Having identified an optimal method for unbiased detection of metabolites, we then applied this method to characterize the utilization of glucose and glutamine in central metabolic pathways. The relative abundance of metabolites detected in an untargeted metabolomics assay does not provide information about the direction of metabolic flux through pathways, which is known to be altered in cancer cells. A detailed characterization of glucose and glutamine utilization under baseline conditions will greatly enhance the interpretation of untargeted metabolomics data. In order to trace carbon flux through central metabolic pathways, we incorporated stable-isotopes of glucose or glutamine into cell culture medium for 1 h prior to harvest of MDA-MB-231 cells. Consistent with the expected glycolytic phenotype, cells exposed to labelled U-^13^C-glucose showed consistent labelling of either all, or 0, carbons in each metabolite throughout the glycolytic pathway, as to be expected with a 50% ^12^C-glucose and ^13^C-glucose mixture (Figure 5, Supplementary Tables S1 and S2). Somewhat unexpectedly, 3-carbon labelled isotopologues of d-fructose 6-phosphate (5%) and d-fructose 1,6-bisphosphate (21%) were also observed, suggesting some level of gluconeogenic flux remains in these cells.

Extensive labelling was also observed in the pentose phosphate pathway (PPP), with complete (6-carbon) labelling of 6-phosphogluconate confirming flux through the oxidative PPP. d-ribose 5-phosphate (R5P) was found to have multiple isotopologues where 2, 3 and 5 carbons were labelled (Figure 5), suggesting that the primary source of R5P is via the non-oxidative branch of the PPP, which can generate 2-, 3- and 5-carbon labelled isotopologues due to the actions of transaldolase and transketolase combining fully labelled and unlabelled precursors [35].

Both glucose and glutamine were incorporated into TCA cycle metabolites. Citrate was predominantly labelled by glucose (76% labelled, corrected for initial glucose enrichment), with the predominant 2-carbon isotopologue indicating that glycolysis-derived pyruvate is metabolized to acetyl CoA, where it combines with oxaloacetate to form citrate. Measurable levels of 3-carbon labelled citrate confirms the role of pyruvate carboxylase in producing some oxaloacetate from glycolytic pyruvate [36]. Whilst oxaloacetate itself could not be measured with this methodology, the 3-carbon labelling observed in aspartate (the transamination product of oxaloacetate) is consistent with pyruvate carboxylase activity. Minimal labelling was observed for the 4- and 5-carbon labelled isotopologues of citrate, indicating that the TCA cycle is not operating as a full cycle, which would be required to generate these higher labelled isotopologues. Indeed, once the cycle reached 2-oxoglutarate, glutamine became the main carbon source (20% glucose, 53% glutamine), and the lower part of the TCA cycle between 2-oxoglutarate and malate appears primarily driven by glutaminolysis. While glutamine-derived oxaloacetate was not detected, we detected 4-carbon labelled isotopologues of aspartate and citrate (28%) derived from labelled glutamine. Collectively, these findings suggest that both glucose and glutamine are important metabolic precursors in these rapidly-dividing cells.

**Figure 6:**
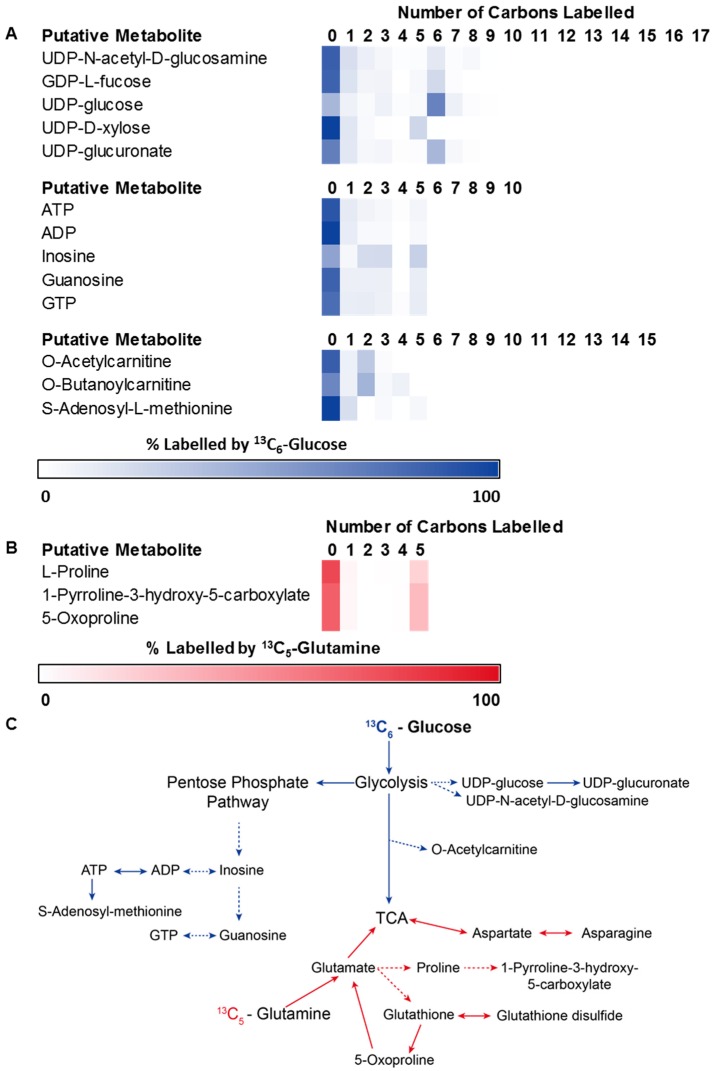
Metabolome-wide detection of isotope-labelled metabolites arising from glucose and glutamine. Heat-map shows the percentage ^13^C-enrichment of each isotopologue for metabolites with >10% ^13^C isotope enrichment. (**A**) Metabolites labelled from U-^13^C-glucose are associated with sugar-nucleotides, nucleotide metabolism and amino acid metabolism. Scale: white represents 0% labelling and blue represents 100% labelling; (**B**) Additional metabolites labelled from U-^13^C-glutamine were limited to amino acid metabolism. Scale: white represents 0% labelling and red represents 100% labelling. (**C**) Schematic representing the pathways involving labelled metabolites from U-^13^C-glucose and U-^13^C-glutamine. (UDP: uridine diphosphate, GDP: guanosine diphosphate, ATP: adenosine triphosphate, ADP: adenosine diphosphate, GTP: guanosine triphosphate).

In addition to the thorough investigation of central metabolic pathways, an untargeted isotope analysis was performed in order to detect flux into other metabolic pathways that branch out from the primary glycolytic and glutaminolytic pathways. The 1-h labelling period did not produce widespread labelling in related pathways, suggesting that longer periods of incubation would be necessary to trace glucose and glutamine utilization throughout the metabolic network. Nevertheless, significant labelling (>10%) was observed in several pathways. Glucose-derived carbon was incorporated into sugar nucleotides from glycolytic intermediates (hexose phosphates), nucleotides from PPP products (R5P) and acetylcarnitine from pyruvate-derived acetyl-CoA (Figure 6A). Glutamine-derived ^13^C labelling was observed in proline and putative 1-pyrroline-3-hydroxy-5-carboxylate, indicating de novo proline synthesis via glutamate (Figure 6B,C). Significant labelling was also observed in the metabolite putatively annotated as 5-oxoproline, which is involved in glutathione recycling. Some labelling was observed in glutathione (3%) and glutathione disulphide (5%), confirming low levels of active glutathione biosynthesis from glutamate (Figure 6B,C).

### 3.3. Application 2: Metabolic Impact of Isoproterenol Treatment

**Figure 7:**
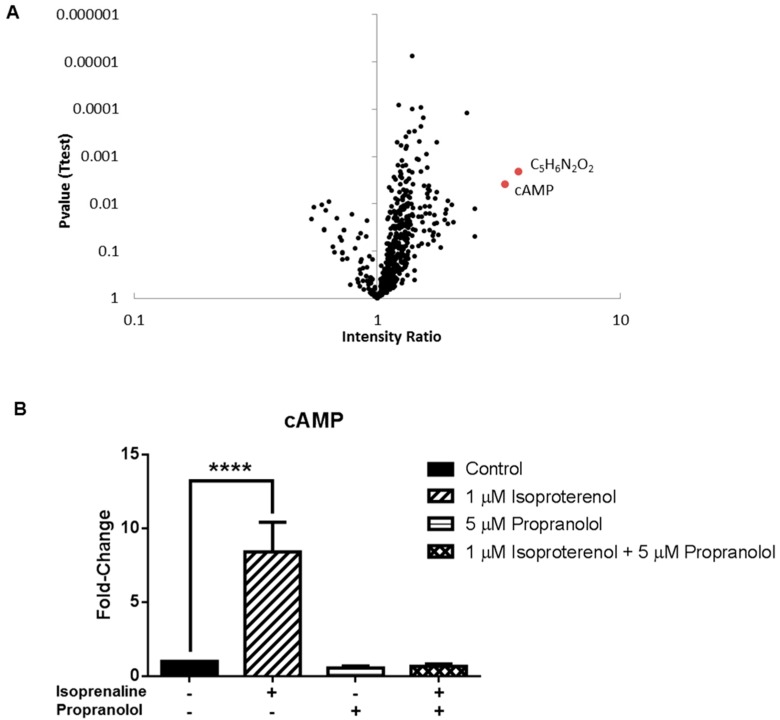
Detection of cAMP accumulation in untargeted metabolomics analysis. (**A**) Volcano plot showing metabolite abundance (LC-MS peak height) relative to untreated controls (*x*-axis) and *p*-value from Welch’s test (*y*-axis) for all metabolites (black circles) in MDA-MB-231 cells treated with isoproterenol for 4 h, compared to untreated control cells. Metabolites with a fold-change >3 and *p*-value <0.05 are shown in red (**B**) cAMP abundance from cells treated with isoproterenol and/or propranolol for 4 h shown as LC-MS peak area relative to untreated control cells (mean ± SE, *n* = 4). **** *p* ≤ 0.0001, using one-way ANOVA with Dunnett’s multiple comparison test.

A critical feature of any untargeted metabolomics method is the ability to detect specific changes in metabolite abundance induced by a defined exposure. To investigate this, we treated cells with isoproterenol, a β-adrenergic receptor agonist, and examined cellular metabolite levels using the one-step in-plate method. β-adrenergic receptors are highly expressed by MDA-MB-231 cancer cells and β-adrenergic receptor signalling drives cancer progression [37,38,39]. β-adrenergic receptor activation results in a conformational change to the receptor, which activates G_αS_ and increases cellular levels of cAMP, triggering other intracellular signalling pathways. Multivariate analysis of the total of 474 putatively identified metabolites, using principal components analysis, revealed distinct separation of the isoproterenol-treated and untreated samples, which was partly reversed by propranolol (Figure S1). Whilst changes to most metabolite levels were subtle (less than 2-fold), univariate analysis (volcano plot) identified significant accumulation of two specific metabolites, cAMP (3.4-fold; *p* = 0.004) and a putative metabolite with monoisotopic mass (126.043) indicating the formula C_5_H_6_N_2_O_2_ (3.8-fold; *p* = 0.002) (Figure 7A). The detection of cAMP accumulation was confirmed in two more independent experiments, and this effect was blocked by pre-treatment with the β-adrenergic receptor antagonist propranolol, demonstrating that the effects are mediated by the β-adrenergic receptor (Figure 7B). The accumulation of the other putative metabolite (C_5_H_6_N_2_O_2_) was not reversed by propranolol, and was not observed in the other two experiments, indicating that this differential feature was likely due to a specific artefact or contamination in one experiment. Importantly, these data show that this optimized untargeted metabolomics method is capable of reproducibly identifying a specific metabolic change, i.e., accumulation of cAMP, among almost 500 putative metabolites following β-adrenergic receptor activation.

## 4. Discussion

These results demonstrate the validation and application of a one-step in-plate extraction method that provides optimal identification and relative quantification of a wide range of metabolites with greater sensitivity than other tested methods. The simplicity and reproducibility of this method provides a standard procedure that will allow comparative untargeted metabolomics studies to detect metabolic changes in diverse pathways, following exposure to specific interventions. Furthermore, the baseline data generated regarding central carbon flux will assist in the design and biochemical interpretation of untargeted metabolomics studies that seek to provide a greater understanding of MDA-MB-231 cell metabolism.

The “one-step in-plate” method involved simultaneous quenching of metabolism and complete extraction of metabolites and provided better coverage than removing the cells from the plate before, or during, extraction. The potential disadvantages of this method were: (i) the need to use a relatively large volume of extraction solvent, which diluted the metabolite concentration; (ii) the necessity for a solvent-resistant cell culture dish; and (iii) the inability to accurately determine the cell density of the sample at the time of extraction. However, these issues were easily overcome by: (i) drying down the extract under nitrogen gas followed by reconstitution for analysis; (ii) using fibronectin-coated glass culture dishes; and (iii) ensuring tightly defined culture conditions and including an additional culture dish for each condition to accurately count the cell density. These minor issues were clearly outweighed by the advantages of rapid in-plate quenching, comprehensive cell lysis and metabolite extraction with a mixed chloroform/methanol/water solvent system. Technical limitations inherent in the “in-tube” and “two-step” methods likely limited their capacity to detect a range of metabolites. For the “in-tube” method, it is likely that the use of a cell scraper to lift cells in saline resulted in cell lysis, releasing polar metabolites into the saline, which was discarded. Thus few polar metabolites remained in the pellet, leading primarily to detection of lipids from cellular membranes. In contrast, the “two-step in-plate” extraction method performed poorly for lipid extraction, as cells were lysed into a methanol and water mixture, where some of the lipids were poorly solubilized and remained in the culture dish. This two-step procedure also introduced more experimental variance than the other methods (Figure 4). We propose that the superior performance of the “one-step in-plate” extraction method was due to the direct solubilization of both polar and non-polar metabolites into the monophasic chloroform/methanol/water solvent. It is possible that the cell adherence properties in the fibronectin-coated glass dish may have also influenced the extraction efficiency, however this is unlikely as there was no difference in cell growth between the two culture dishes. The ability of the one-step in-plate extraction method to detect metabolites with diverse physicochemical properties, and with greater recovery and precision than the other tested methods, makes this method ideal for untargeted metabolomics studies in MDA-MB-231 cancer cells.

Dysregulation of central metabolic pathways, including glycolysis and the TCA cycle, are a hallmark of cancer cell metabolism [40]. It was therefore important to demonstrate the suitability of the optimized untargeted metabolomics method for analysis of these central pathways. Whilst many intermediates of glycolysis and the TCA cycle were detected in the untargeted analysis, the metabolic architecture of these pathways cannot be understood with untargeted metabolomics alone [41], and an understanding of the metabolic flux through central metabolism pathways is essential for interpretation of experimentally-induced perturbations in metabolite levels. Through the use of stable isotope labelling, the activity of key central metabolic pathways was elucidated. Labelling the cells with a 50% mixture of U-^13^C-glucose and U-^12^C-glucose allowed detection of metabolites that were derived from the labelled carbon source, and measurement of the number of carbons labelled in each isotopologue, and the percentage enrichment of each isotopologue, allowed inference of the primary pathways responsible for generation of each metabolite. Glucose was incorporated into the glycolytic pathway as expected [42] and complete labelling of the intracellular pools of glycolytic intermediates within 1 h showed that the pathway was highly active, although accurate quantification of glycolytic flux was beyond the scope of this study. Surprisingly, despite the extensive reliance of cancer cells on glycolysis, the significant levels of 3-carbon labelled fructose 1,6-bisphosphate suggested the presence of some degree of gluconeogenic flux through the reversible action of aldolase. Both the oxidative and non-oxidative branches of the pentose phosphate pathway were also highly active [43], and the labelling pattern of ribose 5-phosphate revealed that the non-oxidative PPP is the primary source of this key nucleotide precursor, consistent with previous studies in pancreatic adenocarcinoma cells [44]. The incorporation of two, and three, carbons from glucose into citrate confirmed the activity of pyruvate dehydrogenase, and pyruvate carboxylase [36], respectively, linking glycolysis to the TCA cycle. Indeed, despite the well-characterized shift away from TCA cycle activity towards glycolysis for energy generation [3], glucose-derived precursors remain responsible for three-quarters of the synthesized citrate. However, the lack of complete cycling of carbon skeletons around the TCA cycle support a primarily anaplerotic, rather than bioenergetic, role for TCA cycle metabolism [45].

Significant utilization of glutamine by MDA-MB-231 was also observed, primarily via conversion to glutamate, which is required for cancer cell proliferation and survival [46]. Glutamine was the predominant carbon source for the part of the TCA cycle linking 2-oxoglutarate to oxaloacetate. This utilization of glutamine for anaplerosis of the TCA cycle is yet another cancer phenotype which is suggested to be important for cell proliferation and survival [47].

Low levels of incorporation of ^13^C-labelled carbon from glucose and glutamine into metabolic pathways beyond central carbon metabolism were also detected. Labelled carbons from glucose were found to be incorporated into UDP-sugars, nucleotides and fatty acyls, which all have established roles in cancer cell proliferation, invasion, metastasis and angiogenesis [48,49]. The limited enrichment of glucose-derived carbon-13 in metabolites beyond glycolysis, PPP and the TCA cycle suggests that the 1 h incubation was not a sufficient duration to reach isotopic steady state in the peripheral pathways, indicating that metabolite turnover rates in those pathways are substantially slower. This finding has implications for the design and interpretation of studies that seek to investigate perturbation of metabolism beyond the high-flux central pathways. However, further studies are required to determine whether the relatively lower isotope enrichment is (partly) due to utilization of alternate carbon sources in these pathways. The methodology described here is ideally suited to further investigation of nutrient utilization in cancer cells, and is likely to provide additional mechanistic information for metabolomics studies that seek to investigate the impact of specific exposures, or interventions, on breast cancer cells.

Lastly, the optimized method was successfully applied in a standard untargeted metabolomics study design to reveal the metabolic impact of a pharmacological intervention with a known response. The activation of β-adrenergic receptors by isoproterenol to stimulate cAMP production, and inhibition of this activity by propranolol, is well characterized [39,50]. The optimized method successfully differentiated treated and untreated cells by multivariate analysis, demonstrating that the intervention had a greater impact on metabolite levels than the inter-day variability associated with the extraction method. Most importantly, the approach successfully identified cAMP accumulation, among almost 500 putative metabolites, as the most significant metabolic perturbation associated with β-adrenergic receptor activation, and this significant cAMP accumulation was confirmed across three independent studies.

## 5. Conclusions

This work describes the development and application of a rapid and efficient extraction method that was suitable for extraction of both polar and lipid metabolites from MDA-MB-231 breast cancer cells for untargeted metabolomics analysis using LC-MS. The method was successfully applied to characterize the metabolism of stable-isotope labelled glucose and glutamine and elucidate the most active metabolic pathways in these cells under standard culture conditions. In addition, we provide “proof-of-concept” that this method is suitable for the unbiased detection of a specific known response caused by pharmacological intervention. These techniques provide a powerful tool for further understanding the role of metabolism in the progression and management of triple negative breast cancer.

## Supplementary Materials

The following are available online at http://www.mdpi.com/2218-1989/6/4/30/s1. Figure S1: Principal components analysis scores plot for the first two principal components from untargeted metabolomics analysis of MDA-MB-231 cells treated for 4 h with isoproterenol (Iso), propranolol (Prop), combination of isoproterenol and propranolol (Prop + Iso) or untreated control cells (control). Values in parenthesis indicate the percentage of variance explained by each principal component, Table S1: Glucose Labelling, Table S2: Glutamine Labelling.

## Abbreviations

The following abbreviations are used in this manuscript:

βAR	β-adrenergic receptor
µL	microliter
µM	micromolar
µm	micrometer
ACN	acetonitrile
ADP	adenosine diphosphate
ATP	adenosine triphosphate
cAMP	3′,5′-cyclic adenosine monophosphate
CO_2_	carbon dioxide
CoA	coenzyme A
DMEM	Dulbecco’s Modified Eagle’s Medium
EDTA	ethylenediaminetetraacetic acid
FBS	fetal bovine serum
g	g-force
GC-MS	gas chromatography mass spectrometry
KV	kilovolt
LC-MS	liquid chromatography mass spectroscopy
mL	milliliter
mm	millimeter
mM	millimolar
MS	mass spectrometry
*m/z*	mass to charge ratio
NMR	nuclear magnetic resonance
PKA	Protein kinase A
ppm	parts per million
PPP	Pentose Phosphate Pathway
QC	quality control
Rpm	revolutions per minute
TCA cycle	tricarboxylic acid cycle
UDP	uridine diphosphate
V	volt
