# Supplementary material for: Optimized Method for Untargeted Metabolomics Analysis of MDA-MB-231 Breast Cancer Cells
Source: Metabolites. 2016 Sep 22;6(4):30. doi: 10.3390/metabo6040030 (PMC5192436; doi:10.3390/metabo6040030)
Supplement: Supplementary file 1 [file metabolites-06-00030-s001.pdf]

Supplementary Materials: Optimized Method for Untargeted Metabolomics Analysis of MDA-MB-231 Breast Cancer Cells

Amanda L. Peterson, Adam K. Walker, Erica K. Sloan and Darren J. Creek

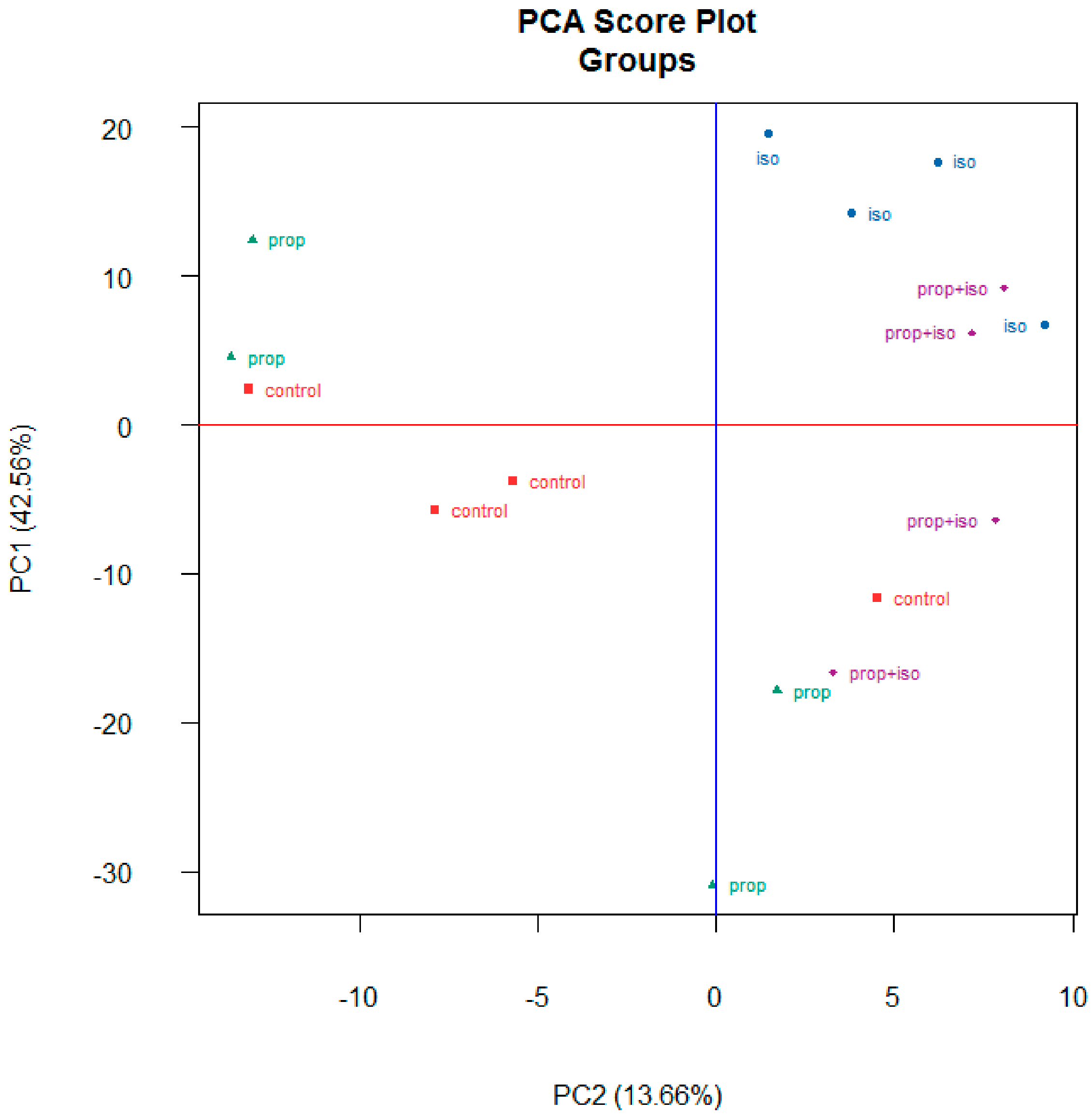

Figure S1. Principal components analysis scores plot for the first two principal components from untargeted metabolomics analysis of MDA-MB-231 cells treated for 4 h with isoproterenol (Iso), propranolol (Prop), combination of isoproterenol and propranolol (Prop + Iso) or untreated control cells (control). Values in parenthesis indicate the percentage of variance explained by each principal component.

Table S1. Carbon-13 isotope enrichment of metabolites in cultured MDA-MB-231 cells following incubation with 50% U-^{13}C-glucose.

Metabolite	%Carbons Labelled																	
	**Total**	**0**	**1**	**2**	**3**	**4**	**5**	**6**	**7**	**8**	**9**	**10**	**11**	**12**	**13**	**14**	**15**	**16**	**17**
D-Glucose $C_6H_{12}O_6$	52%	44%	3%	0%	0%	0%	1%	52%											
D-Glucose 6-phosphate $C_6H_{13}O_9P$	50%	46%	3%	0%	3%	0%	1%	46%											
D-Fructose 6-phosphate $C_6H_{13}O_9P$	44%	53%	4%	0%	5%	0%	1%	37%											
D-Fructose 1,6-bisphosphate $C6H_{14}O_{12}P_2$	61%	36%	3%	0%	21%	1%	2%	37%											
D-Glyceraldehyde 3-phosphate $C_3H_7O_6P$	36%	62%	2%	1%	36%														
Glycerone phosphate $C_3H_7O_6P$	50%	48%	2%	1%	48%														
3-Phospho-D-glycerate $C_3H_7O_7P$	49%	49%	2%	1%	48%														
Phosphoenolpyruvate $C_3H_5O_6P$	49%	49%	2%	3%	46%														
(*S*)-Lactate $C_3H_6O_3$	36%	62%	3%	1%	35%														
6-Phospho-D-gluconate $C_6H_{13}O_{10}P$	52%	45%	3%	0%	7%	0%	2%	42%											
D-Ribose 5-phosphate $C_5H_{11}O_8P$	71%	28%	3%	21%	22%	2%	25%												
Citrate $C_6H_8O_7$	38%	58%	5%	25%	8%	2%	3%	0%											
Isocitrate $C_6H_8O_7$	38%	58%	5%	25%	8%	2%	3%	0%											
2-Oxoglutarate $C_5H_6O_5$	9%	86%	5%	7%	1%	0%	0%												
D-Glutamate $C_5H_9NO_4$	9%	86%	5%	7%	1%	0%	0%												
(*S*)-Malate $C_4H_6O_5$	15%	81%	4%	4%	10%	0%													
L-Aspartate $C_4H_7NO_4$	14%	82%	4%	4%	10%	0%													
UDP-*N*-acetyl-D-glucosamine $C_{17}H_{27}N_3O_{17}P_2$	27%	61%	13%	7%	3%	1%	2%	9%	1%	3%	0%	0%	0%	0%	0%	0%	0%	0%	0%
GDP-L-fucose $C_{16}H_{25}N_5O_{15}P_2$	29%	59%	12%	5%	5%	0%	3%	15%	1%	0%	0%	0%	0%	0%	0%	0%	0%	0%	
UDP-glucose $C_{15}H_{24}N_2O_{17}P_2$	67%	28%	5%	2%	5%	1%	2%	48%	6%	1%	1%	0%	0%	0%	0%	0%			
UDP-D-xylose $C_{14}H_{22}N_2O_{16}P_2$	18%	71%	10%	2%	0%	0%	16%	0%	0%	0%	0%	0%	0%	0%	0%				
UDP-glucuronate $C_{15}H_{22}N_2O_{18}P_2$	42%	49%	9%	3%	4%	1%	1%	28%	3%	1%	0%	0%	0%	0%	0%	0%			
ATP $C_{10}H_{16}N_5O_{13}P_3$	16%	75%	10%	6%	4%	1%	5%	0%	0%	0%	0%								
ADP $C_{10}H_{15}N_5O_{10}P_2$	8%	82%	9%	3%	3%	0%	3%	0%	0%	0%	0%								
Inosine $C_{10}H_{12}N_4O_5$	54%	41%	4%	16%	17%	0%	21%	0%	0%	0%	0%								
Guanosine $C_{10}H_{13}N_5O_5$	22%	70%	7%	7%	7%	0%	9%	0%	0%	0%	0%								
GTP $C_{10}H_{16}N_5O_{14}P_3$	28%	65%	9%	9%	7%	1%	9%	0%	0%	0%	0%								
O-Acetylcarnitine $C_9H_{17}NO_4$	25%	68%	7%	24%	2%	0%	0%	0%	0%	0%									
O-Butanoylcarnitine $C_{11}H_{21}NO_4$	42%	52%	7%	33%	3%	6%	0%	0%	0%	0%	0%	0%							
S-Adenosyl-L-methionine $C15H_{22}N_6O_5S$	7%	79%	15%	0%	3%	0%	3%	0%	0%	0%	0%	0%	0%	0%	0%	0%			
Succinate $C_4H_6O_4$	3%	93%	5%	2%	0%	0%													

Table S2. Carbon-13 isotope enrichment of metabolites in cultured MDA-MB-231 cells following incubation with U-^{13}C-glutamine.

**Metabolite**	**%Carbons Labelled**																					
	**Total**	**0**	**1**	**2**	**3**	**4**	**5**	**6**	**7**	**8**	**9**	**10**	**11**	**12**	**13**	**14**	**15**	**16**	**17**	**18**	**19**	**20**
Citrate $C_6H_8O_7$	26%	69%	5%	2%	3%	17%	4%	0%														
2-Oxoglutarate $C_5H_6O_5$	46%	51%	3%	0%	3%	1%	42%															
L-Glutamate $C_5H_9NO_4$	46%	51%	3%	0%	3%	1%	41%															
(*S*)-Malate $C_4H_6O_5$	28%	69%	3%	0%	3%	25%																
L-Aspartate $C_4H_7NO_4$	27%	70%	3%	0%	3%	24%																
5-Oxoproline $C_5H_7NO_3$	30%	66%	4%	0%	0%	1%	29%															
L-Proline $C_5H_9NO_2$	21%	75%	4%	0%	1%	1%	19%															
L-1-Pyrroline-3-hydroxy-5-carboxylate $C_5H_7NO_3$	30%	66%	4%	0%	0%	1%	29%															
Glutathione disulfide $C_{20}H_{32}N_6O_{12}S_2$	7%	74%	19%	0%	0%	0%	5%	1%	0%	0%	0%	0%	0%	0%	0%	0%	0%	0%	0%	0%	0%	0%
Glutathione $C_{10}H_{17}N_3O_6S$	4%	86%	10%	0%	0%	0%	3%	0%	0%	0%	0%	0%										
Succinate $C_4H_6O_4$	21%	75%	4%	1%	0%	19%																
